# Microbiome Shift, Diversity, and Overabundance of Opportunistic Pathogens in Bovine Digital Dermatitis Revealed by 16S rRNA Amplicon Sequencing

**DOI:** 10.3390/ani10101798

**Published:** 2020-10-03

**Authors:** Hector M. Espiritu, Lovelia L. Mamuad, Seon-ho Kim, Su-jeong Jin, Sang-suk Lee, Seok-won Kwon, Yong-il Cho

**Affiliations:** 1 Department of Animal Science and Technology, Sunchon National University, Suncheon, Jeonnam 57922, Korea; 1193001@s.scnu.ac.kr (H.M.E.); loveliamamuad2306@gmail.com (L.L.M.); mhs0425@daum.net (S.-h.K.); herodias@scnu.ac.kr (S.-j.J.); rumen@scnu.ac.kr (S.-s.L.); 2 Woosarang Animal Hospital, Yongin, Gyeonggi 17026, Korea; gastra00@naver.com

**Keywords:** bovine digital dermatitis, cattle lameness, microbiome, *Treponema* spp

## Abstract

**Simple Summary:**

Bovine digital dermatitis (BDD) is a foot infection known as the primary cause of lameness in cattle due to painful lesions, posing serious impacts on the productivity and welfare of affected animals. Members of the bacterial group *Treponema* have long been considered as the main causative agents because previous investigations by bacterial isolation, tissue analyses, and high molecular sequencing have persistently identified this group in BDD. However, other studies indicated that the presence of several bacteria on the lesion due to the slurry environment the cattle foot are exposed to, suggests an interdependent polybacterial nature which could also play a role in disease development and progression. Therefore, we analyzed the diversity and relationship of the diverse microbiome in BDD lesions compared to normal skin from cattle foot by using next-generation high throughput sequencing. Based on the results obtained, we concluded that the shift in microbial composition which leads to richer diversity in BDD, and the overabundance of opportunistic bacterial pathogens could be associated with BDD pathogenesis.

**Abstract:**

This study analyzed the diversity and phylogenetic relationship of the microbiome of bovine digital dermatitis (BDD) lesions and normal skin from cattle foot by using 16S rRNA amplicon sequencing. Three BDD samples and a normal skin sample were pre-assessed for analysis. The Illumina Miseq platform was used for sequencing and sequences were assembled and were categorized to operational taxonomic units (OTUs) based on similarity, then the core microbiome was visualized. The phylogeny was inferred using MEGA7 (Molecular evolutionary genetics analysis version 7.0). A total of 129 and 185 OTUs were uniquely observed in normal and in BDD samples, respectively. Of the 47 shared OTUs, 15 species presented increased abundance in BDD. In BDD and normal samples, Spirochetes and Proteobacteria showed the most abundant phyla, respectively, suggesting the close association of observed species in each sample group. The phylogeny revealed the evolutionary relationship of OTUs and the Euclidean distance suggested a high sequence divergence between OTUs. We concluded that a shift in the microbiome leads to richer diversity in BDD lesions, and the overabundance of opportunistic pathogens and its synergistic relationship with commensal bacteria could serve as factors in disease development. The influence of these factors should be thoroughly investigated in future studies to provide deeper insights on the pathogenesis of BDD.

## 1. Introduction

Bovine digital dermatitis (BDD) is known as the most important foot infection causing lameness in cattle [1]. This severe lameness, which is the primary clinical manifestation of BDD caused by painful hyperkeratotic lesions [2], poses serious concerns on the welfare of the affected animals [3]. Serious economic impacts have also been implicated with BDD due to significant milk production losses, poor reproductive performance [4,5], and most extremely, premature culling of the affected animals [6].

The first reported case of BDD was in Italy in 1974 by Chelli and Mortellaro [7], and since then, it has been globally reported reaching an endemic state in many countries [8]. Etiological investigations have identified a variety of bacteria in BDD lesions [9], but advancements in sequencing technology have provided essential information on the identity of associated causal agents [8]. In previous investigations, Spirochetes are considered to be the major pathogen in BDD [1,10]. However, studies indicated that the presence of several bacteria on the lesion due to the slurry environment the cattle foot are exposed to [2], suggests a synergistic polybacterial nature [11] which plays a role in disease development and progression [12].

Many reports have attempted to elucidate the association of the microbiome of BDD towards disease development [11,13,14,15]. Surprisingly, for more than 20 years since it emerged as a major problem in the cattle industry in certain countries, studies on BDD have just recently started in Korea. In a recent report, *Treponema* spp. was established as the dominant pathogen in BDD lesions in Korea [16], but an emphasis on the plethora of bacteria that was observed is still lacking. Although the majority of studies considered that the main pathogen of BDD is *Treponema* spp., its polybacterial nature must also be considered in order to understand its pathogenesis [15]. Therefore, this study analyzed the diversity and phylogenetic relationship of the microbiome of BDD lesions and normal skin from the interdigital space of the cattle using 16S rRNA amplicon sequencing.

## 2. Materials and Methods

### 2.1. Sample Collection

This study was performed following general ethical principles and with the consent of farm owners. The collection of samples was done in Holstein–Friesian cattle in housed dairy farm, and was conducted during hoof cleaning, trimming and treatment performed by a professional veterinarian with years of expertise in the field. Inspection was done by anatomic pathological observation of grossly visible active BDD lesions on the proximal border of interdigital space characterized by the presence of ulceration, with hyperkeratosis and proliferative growth with hair-like projections as described by Zinicola et al. (2015) [17]. For normal skin sample, healthy animals with no sign of lameness and no history of BDD were inspected. After thorough cleaning of the foot surface, lidocaine (2%) was subcutaneously injected around the lesion and a 5-mm punch biopsy was taken from the center of the lesion for BDD, while for normal skin, biopsy sample was taken where BDD most often take place. Samples were washed thoroughly with buffered phosphate saline (pH 7.4) and delivered to the laboratory with ice. A total of 66 pre-assessed active BDD samples were subjected to detection of *Treponema* spp. by genus-specific PCR as described in the results of our previous study [16]. Since 100% of the active BDD lesions are positive in PCR, three samples were randomly selected for metagenomics sequencing. A normal sample was provided for BDD negative control. Samples were categorized as Group A for normal skin sample, and Group B for BDD-infected samples.

### 2.2. Metagenomics Sequencing and Diversity Analysis

The four randomly pre-assessed samples were submitted to Macrogen (Korea) for high-throughput sequencing. DNA samples extracted from lesion and normal sample biopsies were subjected to quality control by Picogreen method before library construction. By targeting the V3-V4 region of the 16S rRNA gene, libraries were constructed and were purified. Sequencing was carried out using Illumina Miseq platform (Illumina, San Diego, CA, USA). The base call binary data produced by Real-Time Analysis (RTA) were converted to FASTQ files by bcl2fastq package (Illumina, San Diego, CA, USA) and were filtered using Scythe (v0.994) (https://github.com/vsbuffalo/scythe) and Sickle (https://github.com/najoshi/sickle) programs to remove adapter sequences. The obtained 16S rRNA sequences were binned into Operational Taxonomic Units (OTUs) based on 97% identity using Quantitative Insights Into Microbial Ecology (QIIME) [18]. The microbiome was visualized using Metagenomics Core Microbiome Exploration Tool (MetaCoMET) [19] using a Biological Observation Matrix format (BIOM) [20] generated using Mothur [21].

### 2.3. Divergence and Phylogenetic Analysis

The phylogeny of the microbiome was inferred by aligning the obtained 16S rRNA sequences of the operational taxonomic units identified using ClustalW [22], and the Newick tree data were obtained from MEGA7 [23] by maximum likelihood method following the general time-reversible model as the fit model for calculating the rate of nucleotide base substitution. The final dendrogram was constructed using Iroki [24]. The divergence distance between OTUs was calculated by Euclidian distance method based on the rate of nucleotide base substitution between OTUs, and presented as a heatmap matrix generated by Heatmapper [25] for the aligned sequences of OTUs using the Euclidean distance method.

## 3. Results

In total, 66 samples pre-assessed from our previous research [16] were used in this study. From these, one normal skin sample (Group A) and three BDD lesions (Group B) were subjected for analysis.

**Figure 1:**
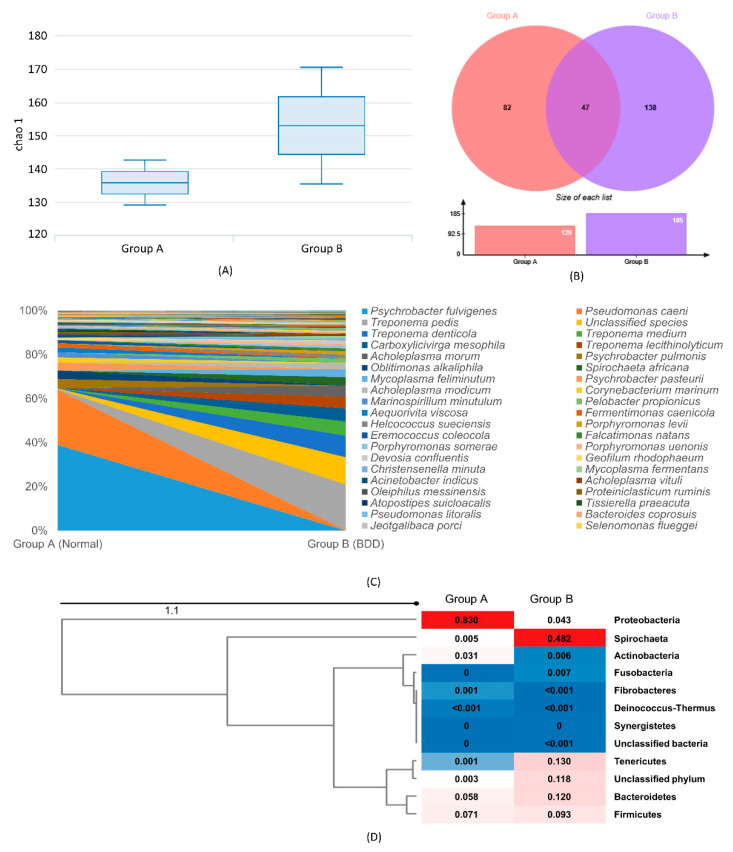
(**A**) Box plot of Chao1 diversity index for bovine digital dermatitis (BDD) (Group A) and normal (Group B), (**B**) the microbiome presenting the number of operational taxonomic units (OTUs) in Group A and Group B showing the unique and shared OTUs between groups, and (**C**) relative abundance of operational taxonomic units from Group A and Group B (legend: 22 most abundant). (**D**) Euclidian distance heatmap presenting the association between phylum and sample groups. Red denotes closer association while blue denotes a lesser association with normal sample and BDD.

The Chao1 diversity index shows that Group B has richer species diversity compared to Group A (Figure 1A). The size of the microbiome presented in Figure 1B shows a higher observed OTUs in BDD lesions compared to the normal skin sample. Out of 267 OTUs observed, 138 were uniquely observed in Group B, 82 in Group A, while 47 were overlapping in both groups. Fifteen of these shared OTUs increased their abundance from normal to BDD and includes T. pedis (20.93%), a group of unclassified species (12.4%), Treponema denticola (9.8%), T. medium (6.48%), Porphyromonas levii (1.56%), P. somerae (1.22%), and Acholeplasma vituli (0.89%). OTUs absent in Group A with increased abundance ratio in Group B were as follows in decreasing order: Carboxylicivirga mesophila (5.89%), T. lecithinolyticum (5.35%), A. morum (5.03%), Spirochaeta africana (3.74%), Mycoplasma feliminutum (3.65%), A. modicum (2.77%), Pelobacter propionicus (2.04%), H. sueciensis (1.64%), P. uenonis (1.30%), Falcatimonas natans (1.25%), Devosia confluentis (1.17), Christensenella minuta (1.08%), and M. fermentans (1.30%). Additional information can be accessed in Supplementary Table S1. The shift in abundance ratio of each species from normal to BDD (Figure 1C) shows the drop in abundance of dominant bacteria, Psychrobacter fulvigenes and Pseudomonas caeni from the normal sample, and the increased abundance of Treponema spp., and other bacteria in BDD. In addition, the Euclidian distance heat-map based on OTU abundance between samples and representative phyla (Figure 1D) illustrated the high association of Spirochetes with BDD. Moraxellaceae and Pseudomonadaceae, both under phylum Proteobacteria presented a close relationship with normal tissue. Moreover, Bacteroidetes and Firmicutes are considerably associated in both groups, while Tenericutes is associated with BDD only.

**Figure 2:**
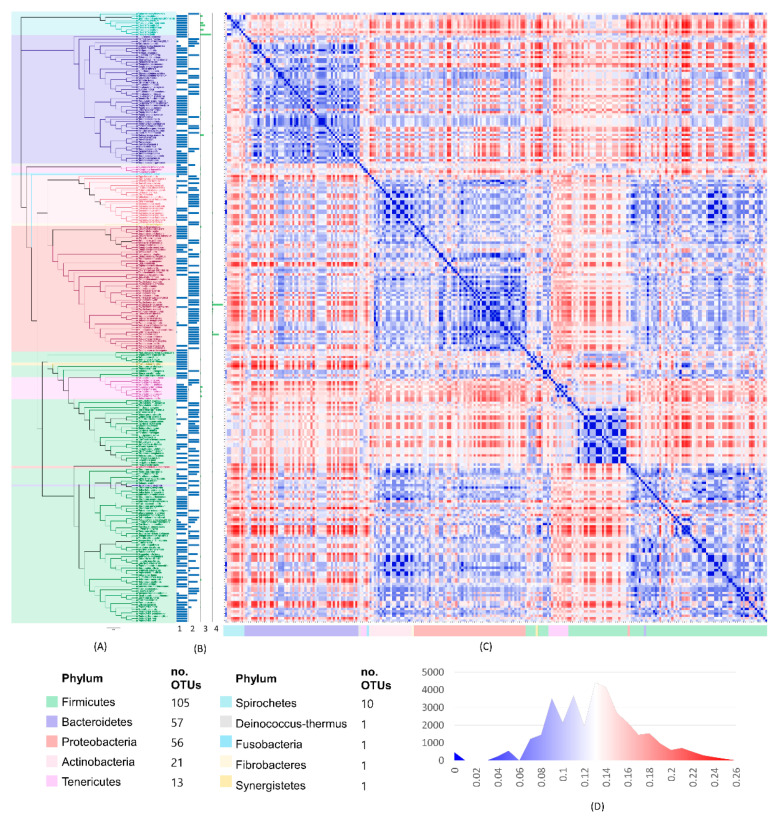
General time-reversible tree representing all observed OTUs in BDD-infected and normal skin samples color-coded based on phylum classification (**A**). The scale value is 2.57. The blue bar graph denotes the abundance ratio of each species on BDD (**B1**) and normal sample (**B2**), while the green bar graph shows the actual count per species (OTU) for BDD (**B3**) and normal (**B4**) sample. Euclidean distance heat map matrix is based on the nucleotide base substitution rate presenting the divergence between all OTUs in BDD and normal sample (**C**). The graph (**D**) shows the frequency distribution of pairwise comparison between OTUs in each calculated distance from low (blue) to high (red).

The maximum likelihood tree constructed using the general time-reversible model illustrated the phylogenetic relationship of all the OTUs (Figure 2A). The tree showed the phylum classification of the OTUs, and the abundance ratio and OTU count (Figure 2B). Firmicutes was the most diverse phylum representing 41.85% and 37.21% of the observed OTUs in BDD and normal skin, respectively. Spirochetes (which is the most abundant) only represented 3.75% of the over-all observed OTUs. In addition, the Euclidean distance heat map matrix showed the estimates of the evolutionary divergence between OTUs in lesions and normal tissue (Figure 2C), and the frequency distribution of the computed distance of the pairwise comparisons was graphed in Figure 2D. Overall, 63.89% of the pairwise comparisons were above the median distance.

## 4. Discussion

This study analyzed the diversity and phylogenetic relationship of the microbiome of BDD lesions compared to normal skin from the interdigital space of the cattle. From our previous study [16], we elucidated the relative abundance of Spirochetes, specifically *Treponema* spp. in BDD lesions and concluded that *Treponema* spp., was the dominant pathogen involved in BDD in Korea. Although the major abundance of *Treponema* spp. in lesions based on several investigations suggest that BDD is polytreponemal, the presence of other bacteria implies that they may also play certain roles in its pathogenesis.

The data analyzed exhibited a change in the microbiome in BDD lesions from the normal skin sample. This altered microbiome has been previously observed by Krull et al., (2014) [13] and Zinicola et al., (2015) [11] by investigating the microbiome of each stage and layer of BDD lesions, respectively. They observed the replacement of species from the normal sample by other species along with the disease progression and the alteration of the microbiome in each layer of lesions. In the current data, the 82 OTUs from the normal sample were replaced in BDD-infected sample by 138 OTUs. This alteration also led to a richer diversity in BDD as opposed to the findings of Krull et al., (2014) where disease progression leads to a drop in diversity [13]. Although samples subjected in our study is categorized only as active BDD, it is recommended to use several normal samples and BDD samples categorized in varying lesion stages to gain more conclusive data. Another interesting finding of this study is the shared OTUs between normal and BDD found in the overlap comprised of three *Treponema* spp. (*T. pedis*, *T. medium*, *T. denticola*), along with two *Phorphyromonas* spp. as the five most abundant bacteria in BDD. The increased abundance of these species from normal to BDD, along with others could imply that these bacteria are commensals which later progresses as opportunistic pathological agents which initiates disease development when triggered under favorable conditions.

The abundance data previously presented by Mamuad et al., (2020) [16], to some extent, agree with the reports of Moreira et al., (2018) [14], Zinicola et al., (2015) [11], Beninger et al., (2016) [26], Krull et al., (2014) [13], and Nielsen et al., (2016) [12], that BDD is polybacterial, with *Treponema* spp., as the most abundant genus. However, the dominance and diversity of species under this genus still varies between reports from different geographical locations. Moreira et al., (2018) reported that *Treponema pedis* is the most abundant species in BDD in Brazil, which is in accordance with Mamuad et al., (2020). However, in one study in the USA, *T. denticola* and *T. phagedenis* were the most abundant species in active and inactive lesions, respectively [11]. Additionally, the results of investigation of Beninger et al., (2015) [26] and Krull et al., (2014) [13] were in agreement with each other that the most abundant species in BDD is *T. phagedenis*, which was absent in all samples in our data. This suggests that BDD microbiome has geographical and/or sample-to-sample variation.

Given that certain members of genus *Treponema* are recognized as the abundant bacteria, and considered as major contributors in the development and progression of the disease, other bacterial genera have also been reported in BDD from other countries as also in this study such as *Porphyromonas* [14], *Campylobacter*, *Acholeplasma* [11], *Peptoniphilus* and *Romboutsia* [27]. In this study, a major abundance of *Carboxylicivirga mesophila* and *Treponema lecithinolyticum* was observed as the fourth and fifth most abundant identified bacteria in BDD, respectively, which were not observed from previous studies. Further investigation is required to verify if these bacteria have actual involvement with the disease, since *C. mesophila* was first isolated from tidal flat sediment in Korea [28], while *T. lecithinolyticum* was commonly found in human oral microbiome [29].

The distance heat map between phyla and samples based on OTU count and taxonomy showed the close relationship of the observed species under phylum Spirochetes and Proteobacteria in BDD and normal skin, respectively. Phylum Bacteroidetes and Firmicutes can be associated in both groups, while Tenericutes is associated only with BDD which agrees with Nielsen et al., (2016) [12]. In a study by Bay et al., (2018) Firmicutes was the most abundant phylum in other polybacterial foot infections in bovine models such as interdigital hyperplasia, interdigital phlegmon, sole ulcer, toe necrosis, and white line disease, with Spirochetes being the fifth most abundant phylum overall, after Bacteroidetes, Actinobacteria, and Proteobacteria [27]. This suggests that there are variations on the major pathogens between these diseases.

The GTR tree shows the phylogenetic relationship of all OTUs present in both BDD-infected and normal skin samples, classified under 10 phyla, with Firmicutes representing the highest diversity. This large number of Firmicutes was supported by the findings of Yano et al., (2010) [30] and Santos et al., (2012) [31] in both normal and BDD-infected samples, regardless of its relative abundance. In this study, Spirochetes with the highest abundance in BDD have lower diversity both before and during infection. Compared with Firmicutes with highest diversity in normal and BDD, there was no increase in its abundance before and during infection. This suggests that diversity richness of a certain phylum may be irrelative with the abundance or its pathogenic involvement in BDD. The microbiome of BDD in this study was verified to be highly diverse, thus, we hypothesize that synergism between overabundant opportunistic pathogens and the diversity of commensals makes the disease more complicated. The pairwise distances of OTUs based on the computed rate of nucleotide substitution show higher frequencies for above-median distances between species found in all samples. This supports the idea that bacterial diversity is evolutionarily diverged.

## 5. Conclusions

We concluded that a shift in the microbiome leads to richer diversity in BDD lesions, and the overabundance of opportunistic pathogens and its possible synergistic relationship between less abundant commensal bacteria could serve as factors in disease development and progression. Spirochetes is the most abundant phylum associated with BDD in other previous studies, and in this study, we deliberated that the abundance of species on each of the observed phylum varied between reports, suggesting either geographical or sample-to-sample variation. The influence of the overabundance of opportunistic species and the synergistic interaction of the plethora of commensal bacteria should be thoroughly investigated in future studies by including additional samples categorized to varying degree of the severity of infection, not just in BDD, but also in other lameness-related foot diseases, to provide deeper insights on the pathogenesis and microbiome relationship of these debilitating diseases.

## Supplementary Materials

The following are available online at https://www.mdpi.com/2076-2615/10/10/1798/s1, Table S1: Abundance ratio of operational taxonomic units in normal and BDD samples.
